# Impact of Non-Payment of Salaries on Treatment Interruption among Patients with Diabetes

**DOI:** 10.31662/jmaj.2025-0279

**Published:** 2025-10-03

**Authors:** Yoshio Shiimoto, Atsushi Goto, Izumi Nakayama, Azusa Arimoto, Takahiro Tabuchi

**Affiliations:** 1 School of Medicine, Yokohama City University, Yokohama, Japan; 2 Department of Public Health, School of Medicine, Yokohama City University, Yokohama, Japan; 3 Department of Health Data Science, Yokohama City University Graduate School of Data Science, Yokohama, Japan; 4 Department of Community Health Nursing, Division of Nursing, Graduate School of Medicine, Yokohama City University, Yokohama, Japan; 5 Division of Epidemiology, School of Public Health, Tohoku University, Graduate School of Medicine, Sendai, Japan

**Keywords:** cohort study, non-payment of salaries, diabetes mellitus, health care access, treatment interruption, COVID-19 pandemic

## Abstract

**Introduction:**

Financial stability during the coronavirus disease 2019 (COVID-19) pandemic was crucial for accessing health care services, including treatment for conditions such as diabetes mellitus. Understanding the relationship between non-payment of salaries and treatment interruption is vital.

**Methods:**

This cohort study investigated the association between non-payment of salaries and treatment interruption in Japanese patients with diabetes. Data from web-based nationwide surveys conducted between July and October 2020 and from January to March 2021 were used.

**Results:**

This study followed up 655 patients with diabetes over time. Of these participants, 15 (2.3%) had recently experienced non-payment for their treatment, whereas 36 (5.5%) had experienced non-payment in the past. Compared with those who received payment for their treatment, the risk ratios (RRs) of treatment interruption were 4.58 (95% confidence interval [CI] 2.34-8.97) for those with recent non-payment and 2.00 (95% CI 0.92-4.36) for those with past non-payment. The RRs for the 548 male participants were 3.67 (95% CI 1.58-8.51) and 2.06 (95% CI 0.88-4.86) for recent and past non-payment, respectively.

**Conclusions:**

In this longitudinal study of Japanese patients with diabetes, we identified an association between non-payment of salary and treatment interruption. Patients who experienced recent non-payment were more likely to experience interruptions in their scheduled care during the COVID-19 pandemic than those who experienced non-payment in the past.

## Introduction

During the coronavirus disease 2019 (COVID-19) pandemic, a notable increase in treatment interruption was observed in patients with type 2 diabetes ^(1), (2), (3)^. Avoiding treatment can result in poor glycemic control ^(4), (5)^, heightening the risk of complications and mortality ^(6), (7)^. Thus, scheduled diabetes treatment is imperative to prevent deteriorating health outcomes ^(8)^.

Understanding the factors that lead to treatment interruptions is essential for its mitigation. Patients may have avoided treatment owing to fear of contracting COVID-19 or fear of visiting health care facilities during the pandemic ^(9)^. In addition, hospitals and clinics may have postponed consultations for patients with COVID-19 to conserve resources ^(10)^. Furthermore, lower income and unemployment were significant factors in treatment interruptions ^(11)^. Notably, many Japanese employees faced salary non-payments due to the pandemic’s economic repercussions ^(12)^, which may have hindered workers from seeking medical consultation. However, to the best of our knowledge, no study has examined whether non-payment of salaries influenced treatment interruptions.

To address this knowledge gap, we conducted a cohort study to investigate whether non-payment is associated with treatment interruptions among patients with diabetes, using Japanese web-based nationwide studies.

## Methods

### Study design and participants

This study is based on data from two internet surveys: the Japan COVID-19 and Society Internet Survey (JACSIS) ^(13)^ and the Japan “Society and New Tobacco” Internet Survey (JASTIS) ^(14)^. The JACSIS was designed to investigate the social and health situation related to the COVID-19 pandemic. The JASTIS is an internet survey focusing on socioeconomic factors and use of heated tobacco products in Japan. The JACSIS 2020 survey was distributed to 224,389 candidates registered as panelists at a Japanese internet research company (Rakuten Insight, Inc., Tokyo, Japan) from August 25 to September 30, 2020, using random sampling stratified by age, sex, and 47 prefectures in Japan. In JASTIS 2021, 26,000 participants were recruited from JACSIS 2020. The JASTIS 2021 survey was distributed to candidates from February 8 to 26, 2021. Participants who provided unreliable responses (i.e., selected “all” in questions regarding the current use of drugs or chronic diseases) were excluded from the analysis.

**Figure 1.**
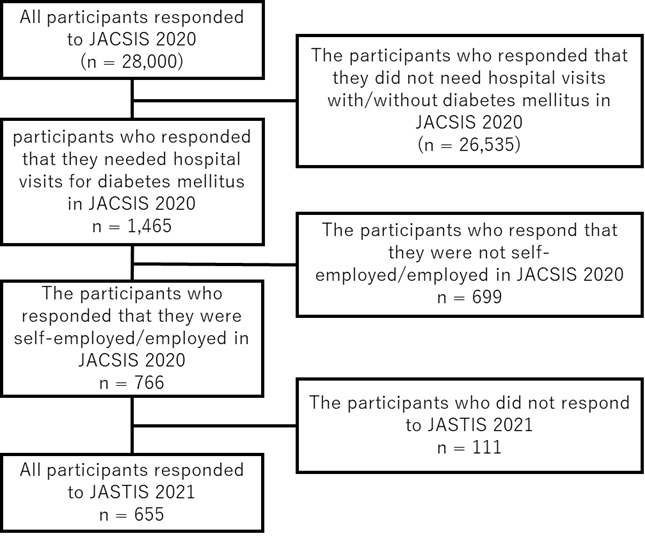
Flow of participants.

Of the 28,000 people (men and women aged 1-79 years) who participated in the JACSIS 2020 survey, 1,465 people who regularly visited medical facilities for diabetes mellitus were included. Of these, 766 individuals who were self-employed/employed in the JACSIS 2020 survey were included. Of 766 individuals, 655 people who responded to the JASTIS 2021 survey were included (Figure 1). Unemployed students, housewives/househusbands, and retired individuals were excluded because they do not receive a salary ^(14)^.

### Measurement

Participants responded to the questionnaire via the internet. In the JACSIS 2020 survey, we evaluated the salary payment status as the exposure of interest. Exposure was classified into three categories (see Supplementary Figure 1 for details):

1. Recent non-payment: those who have experienced non-payment of salary after April 2020.

2. Past non-payment: those who have experienced non-payment of salaries before April 2020.

3. Paid: those who have been duly paid.

The outcome of this study was treatment interruption, as assessed in the 2021 survey. Specifically, we used the following questionnaire item: “Have you experienced any of the following medical or illness-related events in the last two months?” One of the listed events was “being unable to visit or choosing to refrain from attending scheduled medical visits.”

Respondents could select one of three response options: “yes,” “no,” or “not applicable (e.g., no scheduled visit).” A response of yes was classified as indicating a treatment interruption, whereas no or not applicable responses were classified as indicating no interruption.

Socioeconomic factors included age, sex, work status (self-employed, permanently employed, temporarily employed), educational background (non-graduate from university/graduate school, graduate from university/graduate school), and annual household income (<3 million yen, ≥3 million yen, not applicable).

### Statistical analysis

Unadjusted risk ratios (RRs) and multivariable-adjusted RRs for binary dependent variables were estimated using modified Poisson regression with robust standard errors ^(15)^. The multivariable model was adjusted for sex, age, working conditions, educational background, and annual household income. Factors considered as potential effect modifiers were age and sex.

Eighty respondents did not know or did not want to answer about their annual household income. These variables were treated as missing values and then complemented using multiple imputations, using the Multivariate Imputation by Chained Equations (MICEs) package in R ^(16)^. We generated 100 data sets with multiple imputations to account for missing data. Our imputations were based on the predictions made by the variables included in our model. The variables used for imputation included non-payment, treatment interruption, sex, age, working conditions, educational background, and annual household income. The final estimates were then calculated using Rubin’s rule ^(17)^. A complete case analysis was also performed, excluding participants with missing income values. The same analysis was performed for men. As a sensitivity analysis, we repeated the analysis after excluding those who answered not applicable to the question about access to medical facilities in the 2021 survey. All analyses were conducted using R statistical software, version 4.0.5.

## Results

This study included 655 individuals (mean [standard deviation] age, 56.5 [12.8] years; 548 [83.7%] men), of whom 604 (92.2%) reported being paid, 36 (5.5%) reported past non-payment, and 15 (2.3%) reported recent non-payment (Figure 1, Table 1). Patients who did not receive payment in the past tended to be younger, more likely to have a permanent job, more likely to have a lower annual household income, and less likely to be homeowners than those who received payment. Patients who experienced recent non-payment were younger, more likely to have a permanent job, more likely to be university graduates, more likely to have a higher annual household income, and more likely to be unmarried than patients who received payment.

**Table 1.** Baseline Characteristics.

	Category	Paid (n = 604, 92.2%)	Past non-payment (n = 36, 5.5%)	Recent non-payment (n = 15, 2.3%)
Age (mean [SD])		57.78 (11.68)	42.08 (14.62)	41.00 (18.12)
Sex (%)	Male	503 (83.3)	31 (86.1)	14 (93.3)
	Female	101 (16.7)	5 (13.9)	1 (6.7)
Working conditions (%)	Self-employed	108 (17.9)	4 (11.1)	3 (20.0)
	Permanent employed	327 (54.1)	25 (69.4)	9 (60.0)
	Temporary employed	169 (28.0)	7 (19.4)	3 (20.0)
Education (%)	Graduate from university/graduate school	299 (49.5)	17 (47.2)	10 (66.7)
	Non-graduate from university/graduate school	305 (50.5)	19 (52.8)	5 (33.3)
Annual household income (%)	<3 million yen	83 (13.7)	14 (38.9)	2 (13.3)
	≥3 million yen	416 (68.9)	21 (58.3)	13 (86.7)
	Not applicable	105 (17.4)	1 (2.8)	0 (0.0)
Marital status (%)	Married	415 (68.7)	22 (61.1)	8 (53.3)
	Unmarried	132 (21.9)	9 (25.0)	5 (33.3)
	Divorced/widowed	57 (9.4)	5 (13.9)	2 (13.3)
House ownership (%)	Yes	445 (73.8)	21 (58.3)	11 (73.3)
	No	158 (26.2)	15 (41.7)	4 (26.7)

Data are mean (SD), unless otherwise specified. SD: standard deviation.

Table 2 presents the association between those who experienced recent non-payment/past non-payment and treatment interruption. Among the 655 study participants, 58 of 604 (9.6%) who were paid experienced treatment interruption, compared with 8 of 36 (22.2%) with past non-payment and 7 of 15 (46.7%) with recent non-payment. After adjusting for potential confounding factors and imputing missing data using MICE, the RR of treatment interruption was 4.58 (95% confidence interval [CI] 2.34-8.97) for individuals with recent non-payment and 2.00 (95% CI 0.92-4.36) for those with past non-payment, compared with those who received payment for their treatment. Similarly, complete case analysis excluding participants with missing data, indicated an RR of 4.28 (95% CI 2.17-8.46) for recent non-payment and an RR of 1.97 (95% CI 0.90-4.31) for past non-payment.

**Table 2.** Association between Payment and Treatment Interruption.

	Paid		Past non-payment			Recent non-payment		
	N of events/n (%)	RR	N of events/n (%)	RR	95% CI	N of events/n (%)	RR	95% CI
Unadjusted (n = 655)	58/604 (9.6)	1.00 (reference)	8/36 (22.2)	2.31	1.20-4.47	7/15 (46.7)	4.86	2.68-8.80
Multivariable adjusted^a^ (n = 655)	58/604 (9.6)	1.00 (reference)	8/36 (22.2)	2.00	0.92-4.36	7/15 (46.7)	4.58	2.34-8.97
Multivariable adjusted^b^ (n = 549; complete case analysis)	52/499 (10.4)	1.00 (reference)	8/35 (22.9)	1.97	0.90-4.31	7/15 (46.7)	4.28	2.17-8.46

The multivariable model was adjusted for age, sex, education level, annual household income, and working conditions. ^a^Multiple imputation with chained equations. ^b^Complete case analysis: excluding participants who had missing values for income. CI: confidence interval; RR: risk ratio.

Table 3 presents the results for men. Adjusted analyses using MICE showed that the RRs were 3.67 (95% CI 1.58-8.51) for individuals with recent non-payment and 2.06 (95% CI, 0.88-4.86) for those with past non-payment, compared with those who received payment for their treatment. Adjusted complete case analyses showed that the RRs of treatment interruption were 3.53 (95% CI 1.49-8.36) for individuals with recent non-payment and 1.99 (95% CI 0.84-4.74) for those with past non-payment, compared with those who received payment for their treatment.

**Table 3.** Association between Payment and Treatment Interruption for Men.

	Paid		Past non-payment			Recent non-payment		
	N of events/n (%)	RR	N of events/n (%)	RR	95% CI	N of events/n (%)	RR	95% CI
Unadjusted (n = 548)	45/503 (9.0)	1.00 (reference)	8/31 (25.8)	2.89	1.49-5.57	6/14 (42.9)	4.79	2.46-9.33
Multivariable adjusted^a^ (n = 548)	45/503 (9.0)	1.00 (reference)	8/31 (25.8)	2.06	0.88-4.86	6/14 (42.9)	3.67	1.58-8.51
Multivariable adjusted^b^ (n = 471)	41/426 (9.6)	1.00 (reference)	8/31 (25.8)	1.99	0.84-4.74	6/14 (42.9)	3.53	1.49-8.36

The multivariable model was adjusted for age, sex, education level, annual household income, and working conditions. ^a^Multiple imputation with chained equations. ^b^Complete case analysis: excluding participants who had missing values for income. CI: confidence interval; RR: risk ratio.

Supplementary Tables 1 and 2 present the association between recent non-payment and treatment interruption excluding those who answered not applicable to the question about access to medical facilities in the 2021 survey. Adjusted analyses using MICE showed that the RRs were 4.08 (95% CI 2.17-7.69) for those with recent non-payment and 1.97 (95% CI 0.92-4.22) for those with past non-payment, compared with those who received payment for their treatment. For men, the RRs were 3.09 (95% CI 1.30-7.35) for recent non-payment and 1.84 (95% CI 0.74-4.59) for past non-payment.

## Discussion

This longitudinal study investigated the association between non-payment of salaries and treatment interruption among patients with diabetes during the COVID-19 pandemic using nationwide web-based surveys. In this study, 11.1% of workers with diabetes mellitus were unable to visit medical facilities as scheduled. The tendency was higher among those who had recently experienced non-payment of salaries or non-payment of salaries from the past. To the best of our knowledge, this is the first study to analyze the association between non-payment of salaries and visits to medical facilities.

Previous studies have shown that people with low incomes or who are unemployed have difficulty continuing treatment ^(11), (18), (19), (20), (21)^. We found an association between non-payment of salary and an elevated risk of treatment interruption. This suggests that workers who do not receive their salaries may be under financial and psychological pressure, which could potentially lead to treatment interruption. Psychological distress is more likely to result in treatment interruption ^(9)^. Furthermore, economic uncertainty may lead workers to work more instead of actively taking sick leave for fear of a pay cut or losing their jobs ^(22)^. This may prevent them from seeing a medical provider as scheduled. This study also suggests that those who have recently experienced non-payment of salaries may have less access to health care than those who have experienced past non-payment. Sudden non-payment of salaries may not be covered in a timely manner by social security programs, such as financial assistance or exemptions from medical expenses. People in these situations may be reluctant to seek medical care because of the financial burden they may face.

Non-payment of salaries may harm health. Several studies suggest that economically impoverished individuals have health challenges regarding alcohol ^(23), (24)^, smoking ^(24)^, mental health ^(25), (26), (27), (28)^, and self-rated health ^(29), (30)^. Although previous studies have typically focused on the impact of unemployment and low income on individuals, this study has revealed that unpaid salaries can similarly affect access to health care. Moreover, non-payment of salaries can lead to physical and mental health problems, making it highly detrimental to overall health. This study suggests that unpaid wages during the COVID-19 pandemic hampered access to medical care for diabetes patients who require consultation. These findings indicate the need for societal measures to safeguard economically vulnerable individuals during disasters and normal times, thereby reducing health risks.

The findings of this report are subject to at least four limitations. First, our study relied on self-reported data, which are subject to recall, response, and social desirability biases. Second, this study is also subject to a selection bias. The limited sample size and the sample based on two internet surveys, JACSIS and JASTIS, which may not reflect the general Japanese population, may cause selection bias in this study. Moreover, although we adjusted for education level, annual household income, and working conditions, the possibility of residual confounding remains. Third, our results may not be generalizable to other countries with different health care systems. Japan has universal healthcare, but many people in other countries do not have health insurance. In such countries, the financial burden on patients is greater, often leading to treatment interruption. Four, although this study targeted patients with diabetes, it cannot be definitively concluded that they failed to receive diabetes treatment as scheduled. Nevertheless, patients in such circumstances may be proportionately at higher risk of avoiding or deferring diabetes treatment.

In conclusion, in this web-based nationwide longitudinal study of patients with diabetes in Japan, non-payment of salaries was associated with an increased risk of treatment interruption. In particular, workers who recently faced non-payment were less likely to attend scheduled medical visits.

## Article Information

### Acknowledgments

The authors thank all study respondents and lab members for their sincere support. We would like to thank Editage (www.editage.jp) for English language editing.

### Author Contributions

Yoshio Shiimoto analyzed the data and wrote the first draft of the manuscript. Atsushi Goto, Azusa Arimoto, and Takahiro Tabuchi critically revised the manuscript. Atsushi Goto and Takahiro Tabuchi supervised the study. Yoshio Shiimoto, Atsushi Goto, and Takahiro Tabuchi organized the study design. All authors approved the final version of the manuscript.

### Conflicts of Interest

Takahiro Tabuchi received financial support for research (research fundings, consulting fees or lecture fees) from Daiichi Sankyo Healthcare Co., Ltd., Johnson & Johnson K.K., Data Seed Inc., Workout-Plus LLC and EMMA Co., Ltd. (in the last 36 months).

### Ethics Approval and Consent to Participate

The institutional review boards of Yokohama City University and Osaka International Cancer Institute approved this study. The participants were requested to provide web-based informed consent before responding to the online questionnaire. In the cases of minors, defined as individuals below the age of 16 years, informed consent to participate was obtained from the parents or legal guardians. After completing the survey, participants received a credit point known as an “E-point,” which could be used for internet shopping and cash conversion, as an incentive.

### Availability of Data and Materials

The data used in this study cannot be made available in a public repository because it contains personally identifiable and potentially sensitive patient information. In accordance with ethical guidelines in Japan, the Research Ethics Committee of the Osaka International Cancer Institute has placed restrictions on the dissemination of the data collected in this study. For any data inquiries, please contact the individual responsible for data management, Dr. Takahiro Tabuchi, at the following email address: tabuchitak@gmail.com.

## Supplement

Supplementary Material

## References

[1] Aubert CE, Henderson JB, Kerr EA, et al. Type 2 diabetes management, control and outcomes during the COVID-19 pandemic in older US veterans: an observational study. J Gen Intern Med. 2022;37(4):870-7. 34993873 10.1007/s11606-021-07301-7 PMC8735737

[2] Yagome S, Sugiyama T, Inoue K, et al. Influence of the COVID-19 pandemic on overall physician visits and telemedicine use among patients with type 1 or type 2 diabetes in Japan. J Epidemiol. 2022;32(10):476-82. 35691909 10.2188/jea.JE20220032 PMC9424188

[3] Ikesu R, Miyawaki A, Sugiyama T, et al. Trends in diabetes care during the COVID-19 outbreak in Japan: an observational study. J Gen Intern Med. 2021;36(5):1460-2. 33469742 10.1007/s11606-020-06413-w PMC7814982

[4] Kim N, Agostini JV, Justice AC. Refill adherence to oral hypoglycemic agents and glycemic control in veterans. Ann Pharmacother. 2010;44(5):800-8. 20388863 10.1345/aph.1M570 PMC3117591

[5] Wei W, Pan C, Xie L, et al. Real-world insulin treatment persistence among patients with type 2 diabetes. Endocr Pract. 2014;20(1):52-61. 24013990 10.4158/EP13159.OR

[6] Adler AI, Stratton IM, Neil HA, et al. Association of systolic blood pressure with macrovascular and microvascular complications of type 2 diabetes (UKPDS 36): prospective observational study. BMJ. 2000;321(7258):412-9. 10938049 10.1136/bmj.321.7258.412 PMC27455

[7] Khunti K, Seidu S, Kunutsor S, et al. Association between adherence to pharmacotherapy and outcomes in type 2 diabetes: a meta-analysis. Diabetes Care. 2017;40(11):1588-96. 28801474 10.2337/dc16-1925

[8] Davies MJ, Aroda VR, Collins BS, et al. Management of hyperglycemia in type 2 diabetes, 2022. A consensus report by the American Diabetes Association (ADA) and the European Association for the Study of Diabetes (EASD). Diabetes Care. 2022;45(11):2753-86. 36148880 10.2337/dci22-0034 PMC10008140

[9] Minoura A, Sugiyama T, Koyama T, et al. Structural equation modeling of the effects of psychological distress and a fear of coronavirus disease 2019 on diabetes care in Japan: a cross-sectional study. Sci Rep. 2022;12(1):16142. 36167976 10.1038/s41598-022-20716-4 PMC9514688

[10] Ii M, Moriyama M, Watanabe S. Patient behavior during the COVID-19 pandemic and impacts on medical institution revenue. Public Policy Rev. 2023;19:1-39.

[11] Fujimoto K, Ishimaru T, Tateishi S, et al. A cross-sectional study of socioeconomic status and treatment interruption among Japanese workers during the COVID-19 pandemic. J Occup Health. 2021;63(1):e12232. 33998105 10.1002/1348-9585.12232 PMC8126756

[12] Hamada I. Double truth: employment insecurity and gender inequality in Japan’s neoliberal promotion of side jobs. Jpn Forum. 2024;36(3):329-51.

[13] Yoshioka T, Okubo R, Tabuchi T, et al. Factors associated with serious psychological distress during the COVID-19 pandemic in Japan: a nationwide cross-sectional internet-based study. BMJ Open. 2021;11(7):e051115. 10.1136/bmjopen-2021-051115 PMC8260284 34226236

[14] Hori A, Tabuchi T, Kunugita N. Rapid increase in heated tobacco product (HTP) use from 2015 to 2019: from the Japan ‘Society and New Tobacco’ Internet Survey (JASTIS). Tob Control. 2021;30(4):474-5. 10.1136/tobaccocontrol-2020-055652 PMC8237184 32503900

[15] Zou G. A modified Poisson regression approach to prospective studies with binary data. Am J Epidemiol. 2004;159(7):702-6. 15033648 10.1093/aje/kwh090

[16] van Buuren S, Groothuis-Oudshoorn K. Mice: multivariate Imputation by Chained Equations in R. J Stat Soft. 2011;45(3):1-67.

[17] Rubin DB. Multiple imputation for nonresponse in surveys. Chichester, UK: John Wiley & Sons; 2004. 320 p.

[18] McMaughan DJ, Oloruntoba O, Smith ML. Socioeconomic status and access to healthcare: interrelated drivers for healthy aging. Front Public Health. 2020;8:231. 32626678 10.3389/fpubh.2020.00231 PMC7314918

[19] Murata C, Yamada T, Chen CC, et al. Barriers to health care among the elderly in Japan. Int J Environ Res Public Health. 2010;7(4):1330-41. 20617033 10.3390/ijerph7041330 PMC2872331

[20] Driscoll AK, Bernstein AB. Health and access to care among employed and unemployed adults: United States, 2009-2010. NCHS Data Brief. 2012;(83):1-8. 22617552

[21] Devoe JE, Baez A, Angier H, et al. Insurance + access not equal to health care: typology of barriers to health care access for low-income families. Ann Fam Med. 2007;5(6):511-8. 18025488 10.1370/afm.748 PMC2094032

[22] Kinman G, Grant C. Presenteeism during the COVID-19 pandemic: risks and solutions. Occup Med (Lond). 2021;71(6-7):243-4. 33205200 10.1093/occmed/kqaa193 PMC7717418

[23] Jørgensen MB, Pedersen J, Thygesen LC, et al. Alcohol consumption and labour market participation: a prospective cohort study of transitions between work, unemployment, sickness absence, and social benefits. Eur J Epidemiol. 2019;34(4):397-407. 30627937 10.1007/s10654-018-0476-7 PMC6451700

[24] Latif E. The impact of recession on drinking and smoking behaviours in Canada. Econ Modell. 2014;42:43-56.

[25] Cygan-Rehm K, Kuehnle D, Oberfichtner M. Bounding the causal effect of unemployment on mental health: nonparametric evidence from four countries. Health Econ. 2017;26(12):1844-61. 28497638 10.1002/hec.3510

[26] Stankunas M, Kalediene R, Starkuviene S, et al. Duration of unemployment and depression: a cross-sectional survey in Lithuania. BMC Public Health. 2006;6:174. 16822310 10.1186/1471-2458-6-174 PMC1526724

[27] Chang SS, Gunnell D, Sterne JAC, et al. Was the economic crisis 1997-1998 responsible for rising suicide rates in East/Southeast Asia? A time-trend analysis for Japan, Hong Kong, South Korea, Taiwan, Singapore and Thailand. Soc Sci Med. 2009;68(7):1322-31. 19200631 10.1016/j.socscimed.2009.01.010

[28] Frasquilho D, Matos MG, Salonna F, et al. Mental health outcomes in times of economic recession: a systematic literature review. BMC Public Health. 2016;16:115. 26847554 10.1186/s12889-016-2720-y PMC4741013

[29] Heggebø K, Elstad JI. Is it easier to be unemployed when the experience is more widely shared? Effects of unemployment on self-rated health in 25 European countries with diverging macroeconomic conditions. Eur Sociol Rev. 2018;34(1):22-39.

[30] López Del Amo González MP, V Benítez, JJ Martín-Martín. Long term unemployment, income, poverty, and social public expenditure, and their relationship with self-perceived health in Spain (2007-2011). BMC Public Health. 2018;18(1):133. 29334909 10.1186/s12889-017-5004-2 PMC5769359

