# Supplementary material for: Impact of Non-Payment of Salaries on Treatment Interruption among Patients with Diabetes
Source: JMA J. 2025 Oct 3;8(4):1227–33. doi: 10.31662/jmaj.2025-0279 (PMC12598205; doi:10.31662/jmaj.2025-0279)
Supplement: Supplementary Material [file 2433-3298-8-4-1227-s001.pdf]

Supplementary Table 1. Association between Payment and Treatment Interruption Excluding Those Who Answered “Not Applicable” to the Question about Access to Medical Facilities in the 2021 Survey.

	Paid		Past non-payment			Recent non-payment		
	N of events/n (%)	RR	N of events/n (%)	RR	95% CI	N of events/n (%)	RR	95% CI
Unadjusted (n=492)	58/453 (12.8)	1.00 (reference)	8/27 (29.6)	2.31	1.23-4.34	7/12 (58.3)	4.56	2.67-7.78
Multivariable adjusted [a] (n=492)	58/453 (12.8)	1.00 (reference)	8/27 (29.6)	1.97	0.92-4.22	7/12 (58.3)	4.08	2.17-7.69
Multivariable adjusted [b] (n=412; complete-case analysis)	52/373 (13.9)	1.00 (reference)	8/27 (29.6)	1.86	0.87-3.98	7/12 (58.3)	3.81	2.01-7.20

The multivariable model was adjusted for age, sex, education level, annual household income, and working conditions.

[a] Multiple imputation with chained equation

[b] Complete-case analysis: excluding participants who had missing values for income

Confidence interval, CI; risk ratio, RR.

Supplementary Table 2. Association between Payment and Treatment Interruption for Men, Excluding Those Who Answered “Not Applicable” to the Question about Access to Medical Facilities in the 2021 Survey.

	Paid		Past non-payment			Recent non-payment		
	N of events/n (%)	RR	N of events/n (%)	RR	95% CI	N of events/n (%)	RR	95% CI
Unadjusted (n=415)	45/380 (11.8)	1.00 (reference)	8/24 (33.3)	2.82	1.50-5.28	6/11 (54.5)	4.61	2.52-8.44
Multivariable adjusted [a] (n=415)	45/380 (11.8)	1.00 (reference)	8/24 (33.3)	1.84	0.74-4.59	6/11 (54.5)	3.09	1.30-7.35
Multivariable adjusted [b] (n=356; complete-case analysis)	41/321 (12.8)	1.00 (reference)	8/24 (33.3)	1.82	0.73-4.51	6/11 (54.5)	3.08	1.30-7.33

The multivariable model was adjusted for age, sex, education level, annual household income, and working conditions.

[a] Multiple imputation with chained equation

[b] Complete case analysis: excluding participants who had missing values for income

CI: confidence interval; RR: risk ratio.
